# Degradation of gaseous volatile organic compounds (VOCs) by a novel UV-ozone technology

**DOI:** 10.1038/s41598-022-14191-0

**Published:** 2022-06-30

**Authors:** G. Oliva, J. R. Comia, V. Senatore, T. Zarra, F. Ballestreros, V. Belgiorno, V. Naddeo

**Affiliations:** 1 SEED—Sanitary Environmental Engineering Division, Department of Civil Engineering, University of Salerno, Via Giovanni Paolo II, 132, 84084 Fisciano, Salerno Italy; 2 Department of Environmental Engineering, University of the Philippines, Diliman, Quezon City, Metro Manila Philippines

**Keywords:** Pollution remediation, Chemical engineering

## Abstract

In this study, a UV-assisted ozonation (UV/O_3_) process for the degradation of VOCs emissions with a final scrubbing phase was implemented to evaluate the removal efficiency of toluene and to prevent the release of polluting intermediates of the single-step process. Inlet toluene concentration and applied voltage were varied in order to investigate several operating conditions. The results highlighted that at higher inlet concentration the abatement of toluene was lower, while increase in ozone concentration led to an increase of the degradation efficiencies. The additional water scrubbing step enhanced the abatement of UV/O_3_ up to 98.5%, due to the solubilisation of ozone and by-products in the process water and, thus, the further oxidation of the contaminants within this phase. A maximum Elimination Capacity (EC_max_) of 22.6 g m^−3^ h^−1^ was achieved with the UV/O_3_ + Scrubbing. The combined system boosted higher performance and stability compared to the stand-alone (UV/O_3_) process along with a more economical and environmental sustainability.

## Introduction

Volatile organic compounds (VOCs) emissions have become a key environmental concern due to adverse effects on health and environment^1–4^. Extended human exposure to VOCs emissions (e.g., aromatic substances and aldehydes) may cause various health problems such as digestive, kidney, cardiac, and nervous disorders when ingested, in contact with the skin or inhaled^5,6^. Volatile aromatic hydrocarbons, including Benzene, Toluene, Ethylbenzene, and Xylene (BTEX) are classified as toxic, mutagenic, and carcinogenic^7–9^. Due to these characteristics, the BTEX are included within the substances with the greatest human health threat^10,11^.

In the atmosphere, solar radiation triggers VOCs to react with nitrogen oxides, causing photochemical smog and pollution^7,12,13^. For their high volatility, VOC emissions from different industrial sectors are also responsible for odor annoyance among the exposed population. Since that, odor management is becoming a prior issue for industrial operator with a view at complying with regulations and avoiding complaints from resident population^14^. Petrochemical industry, plants involving paints, adhesives, solvents production, printing operations, as well as waste and wastewater treatment plants are among the main sources of anthropogenic emissions of BTEX^15–18^.

The more stringent regulations on air pollution and the higher expectation of the population about air quality trigger the need for the effective management and treatment of these compounds^14,19^. In this view, the scientific community have focused the attention on the development of innovative solutions for their abatement^20,21^.

The abatement techniques currently applied for the VOC emissions treatment involves activated carbon adsorption, absorption, biofiltration and thermal or catalytic combustion^22–24^. The drawbacks of these conventional solutions include contaminant transfer to other phases, inefficient biological treatments due to load fluctuations and presence of recalcitrant and toxic secondary metabolites^25–27^. To overcome the limitations of the conventional processes, numerous researches are focused on Advanced Oxidation Processes (AOPs) for the abatement of VOCs. These processes rely on the effects of hydroxyl radicals- highly reactive oxidants—able to degrade a wide-range of organic compounds^22,28,29^.

Ozonation supported by ultraviolet irradiation (UV/O3) is an established method to degrade recalcitrant and hydrophobic VOCs^30,31^. Ozone enhanced the degradation capability of the combined process not only due to its high oxidation power, but also by the formation of strong oxidants such as hydroxyl and oxide radicals^32^.

The aim of the present work was to evaluate the performances of UV-assisted ozonation (UV/O3) process to enhance VOCs degradation. Toluene was identified as target compound for the experimental activities. A novel configuration with an additional scrubbing phase is proposed and assessed to improve the removal efficiency and to prevent the release of polluting intermediates of the single-step process.

Ozone was produced by the irradiation of air with the UV lamps. The photolysis/irradiation of ozone then leads to the hydroxyl radicals formation^33^. The presence of water vapor irradiated with UV also generate additional radicals^34^. However, the further irradiation of ozone lead to the greater contribution of hydroxyl radicals. Different studies evaluated the performances of toluene oxidation by UV/O_3_, varying different operational parameters, including inlet concentration, ozone concentration, gas flow rate and humidity, using UV lamps and separate ozone generators^3,35^. These studies suggested that the UV/O_3_ process is a suitable technology with the aim at degrading toluene. The main disadvantage of the application of UV/O_3_ treatment is the formation of undesirable by-products resulting from the UV irradiation and ozone oxidation^16^. Since most of the by-products formed by toluene oxidation have high water solubility, the integration with a conventional air bubble wet scrubbing supported a synergic effect. The water-scrubbing step allowed to overcome the main limitation of the stand-alone system, mainly concerning the release of undesirable by-products into the atmosphere and the high energy consumption^34,36^. Advanced Oxidation Process (AOPs) and biological processes support the oxidation of the organic pollutants, promoting their conversion into harmless and odourless compounds. The integration of booth processes is suggested to increase treatability of VOCs^24^. The influence of different operational parameters on the removal of toluene was analysed to define and evaluate the performances of the investigated systems.

## Results

### Influence of inlet concentration

**Figure 1:**
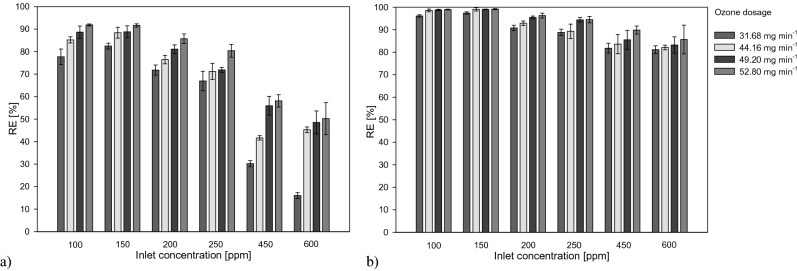
Influence of toluene inlet concentration on the Removal Efficiency (RE) under different ozone dosage applying the UV/O_3_ system (**a**) and the UV/O_3_ + S system (**b**).

Figure 1 illustrates the effect of the increasing in inlet concentration on the degradation of toluene respectively for the UV-Ozone system (UV/O_3_) and the combined system (UV/O_3_ + S), under different ozone dosages.

Applying the lowest ozone dosage, the UV/O_3_ system did not support an effective toluene removal for the highest inlet concentrations. The combined systems achieved, with the same ozone dosage, removal efficiencies higher than 80% for all the investigated inlet concentrations. Removals up to 99% were obtained with the two-stage system. The higher performances of the combined system may be ascribable to the fact that the scrubbing phase promoted the solubilization of the ozone and related by-products into the liquid phase; the residue ozone in the process water carried out a further oxidation of the contaminants transferred to this phase.

**Figure 2:**
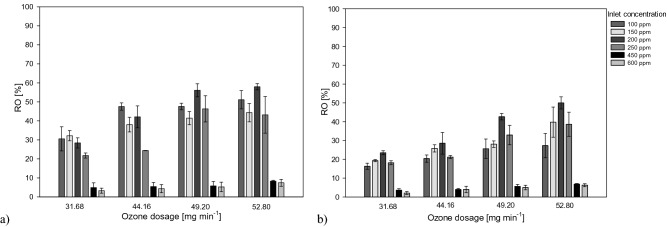
Influence of toluene inlet concentration on the Residue Ozone (RO) under different ozone dosage applying the UV/O_3_ system (**a**) and the UV/O_3_ + S system (**b**).

In Fig. 2 are reported the results in terms of residual ozone.

With the increase in concentrations, the percentages of residual ozone tended to decrease. With an ozone dosage of 31.68 mg min^−1^ and an inlet toluene concentration of 600 ppm, the residual ozone resulted less than 3% for the combined process. The presence of the scrubbing phase, indeed, supported an effective reduction of the residual ozone. This effect resulted more appreciable at lower inlet concentrations. For all the investigated ozone dosages, at 450 and 600 ppm, the residual ozone resulted less than 10% for both investigated systems.

### Influence of ozone dosage

Ozone concentration resulted dependent on the applied voltage. An increase in the applied voltage resulted, obviously, into an increase in ozone dosage. Switching on 1 lamp a corresponding ozone production of 31.7 mg min^−1^ was achieved. With 2 lamps on, it was obtained an ozone dosage equal to 44.16 mg min^−1^. However, the increase in ozone dosage switching on from 3 to 4 lamps resulted in the increase of ozone dosage from 49.2 to 52.8 mg min^−1^. The trend in the increase was, thus, not linear due to the configuration of the lamps in the reactor. The two lamps in the center resulted directly invested by the airflow, but the other two located respectively at the top and bottom part of the reactor received less air flow and, consequently, contributed less to the ozone production.

**Figure 3:**
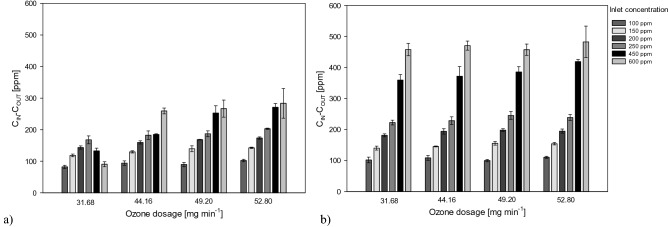
Influence of ozone dosage on the toluene abatement under different inlet concentrations applying the UV/O_3_ system (**a**) and the UV/O_3_ + S system (**b**).

In Fig. 3 are reported the results in terms of toluene reduction (ppm) varying the ozone dosage, for different inlet concentrations.

The maximum toluene reduction for the UV/O_3_ system at 100 ppm was achieved for the maximum ozone dosage and resulted equal to 102 ± 4 ppm; at the same conditions, the combined system supported a toluene removal of 110 ± 4 ppm. For an inlet concentration of 150 ppm, the analysis of the reduction in toluene concentration highlighted the same behavior. At an inlet concentration of 200–250 ppm, the effect of the increasing in the provided ozone started to be more appreciable. At the same time, the combination with the scrubbing unit seemed to promote an enhancement of the performance, for each ozone dosage. At 450 ppm inlet toluene concentration, the eliminated toluene for the lowest ozone dosage resulted equal to 143 ± 5 ppm for the single-step process, while the value providing the maximum ozone dosage reached 174 ± 4 ppm. The combined system allowed to achieve 195 ± 6 ppm toluene removal for an applied a voltage producing 52.8 mg min^−1^ of ozone.

## Discussion

With the increase in inlet concentration, applying the highest ozone dosages (44.16 and 52.80 mg min^−1^), it was observed that the stand-alone system still highlighted removal efficiency significantly reduced for the highest toluene inlet concentrations. This effect might be probably ascribable to the toluene accumulation and the simultaneous insufficiency of oxidants to support the degradation. The following reaction, resulting in the mineralization into carbon dioxide, was not favored due to the limitation in terms of hydroxyl radicals^37,38^. In this case, the scrubbing process ensured a higher transfer of ozone into the liquid phase due to the high solubility of these compounds in water^24^.

The presence of unreacted ozone and toluene was attributed to the faster reaction rate of ozone with hydroxyl radicals (5.3 × 10^–11^ cm^3^ molecule^−1^ s^−1^) compared to the reaction of ozone with toluene (1.2 × 10^–20^ cm^3^ molecule^−1^ s^−1^)^39,40^. The excess ozone may scavenge the hydroxyl radicals, which reacted with O_3_ instead of toluene. These conditions probably led to the incomplete degradation of the pollutants. At high ozone concentrations, it may be observed also a limiting reaction of UV with ozone for radical production. Higher ozone concentration limited the amount of highly reactive radicals and led to the formation of less reactive radicals as also observed by^40,41^. Other Ox-HOx reactions of second-order, indeed, could also occur at high ozone concentrations, reducing the rate of oxidation^42^.

For the lowest inlet concentrations, the increase in ozone dosage did not significantly affected the reduction of toluene. Furthermore, the results of the stand-alone process resulted quite similar to the results of the combined systems.

The toluene elimination increased with the increasing in toluene inlet load a part from what observed for an ozone dosage of 31.68 mg min^−1^ in the single-stage reactor. The results observed at these conditions may be probably due to an effect of toluene accumulation in the reactor. The maximum toluene elimination was achieved for an ozone dosage of 52.8 mg min^−1^ in the two-stage system and resulted equal to 483 ± 51 ppm. For the same operational parameters, the result achieved by the single-stage system was only 283 ± 47 ppm. The ozone and oxidants in the aqueous phase did not represent a limiting factor for the oxidation process in the combined systems, differently by the conditions observed in the single-stage process.

A greater amount of ozone provided a higher quantity of oxidants for a more effective pollutant removal. Based from the experimental data, for the lowest toluene inlet concentrations the effect of the increase in ozone dosage in the oxidation of toluene resulted marginal. This condition may be ascribed to the fact that toluene oxidation by hydroxyl radicals was the predominant mechanism in the process at these conditions. On the other hand, for higher inlet concentrations reactions of oxidants with toluene prevailed as major reaction pathways.

The oxidation of toluene occurs in the gaseous bulk and therefore, in addition to hydroxyl radicals, radical oxygen is also one of the main causes of decomposition. According to the literature, in the UV/O3 process, formic acid, acetic acid, benzyl alcohol, benzaldehyde, p-cresol, hydroquinone, and benzoic acid were observed as the main byproducts. Consequently, the corresponding transformation pathways entails that toluene react rapidly with oxygen species to generate acids which can open the aromatic ring and convert toluene into some less reactive and less toxic intermediate with linear structure.

**Figure 4:**
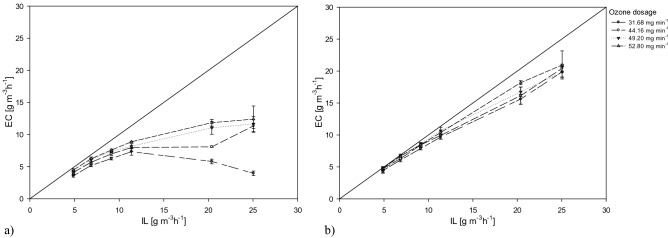
Inlet Load (IL) compared to Elimination Capacity (EC) applying the UV/O3 system (**a**) and the UV/O3 + S system (**b**).

In Fig. 4 it was compared the elimination capacity of both investigated systems to the inlet load with a view at comparing the performances of the UV/O_3_ system and the combined system.

**Figure 5:**
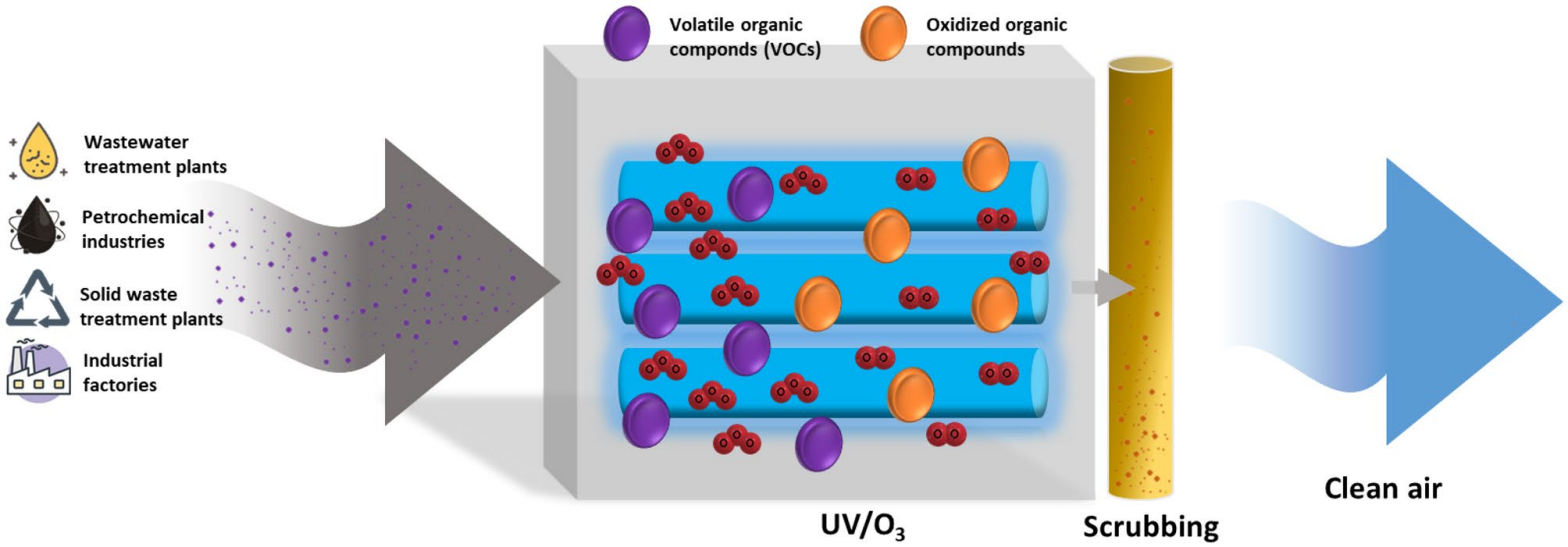
Schematic representation of the advanced oxidation process coupled with wet scrubbing for the VOCs removal from industrial and environmental facilities.

The results highlighted UV/O_3_ + S process supported removal efficiencies closer to the ideal condition of 100% removal. The comparison between the two graphs showed that applying the minimum ozone dose in the combined process were achieved higher treatment efficiencies than those obtained, at all the lamps on, with the single-stage treatment. Increasing the inlet load, with the combined system the elimination capacities remained still close to the ideal condition. In UV/O_3_ process, a significant reduction in toluene removal was observed for toluene inlet load higher than 10 g m^−3^ h^−1^. In the water scrubbing step, in fact, further degradation of unreacted toluene was due to absorption in the water phase and reaction with hydroxyl radicals from ozone decomposition. The scrubbing process provided a venue for the contact and oxidation by hydroxyl radicals in the liquid phase, as reported in Fig. 5. Moreover, the scrubbing increased ozone consumption and lessened the exhaust ozone emissions.

Gas phase toluene degradation by UV irradiation with ozonation was indeed investigated and compared with the process upgraded by an additional step of scrubbing. The increase in inlet toluene concentration led to the drop of the removal efficiency for the single-stage process. The main mechanism of the process was through the oxidation of toluene by hydroxyl radicals. The additional water scrubbing phase facilitated further degradation of toluene and increased the ozone consumption. The UV/O_3_ process supported an effective toluene removal for inlet concentration less than 200 ppm, with 1 lamp on. For greater concentrations, higher voltage is needed to achieve adequate removal. At all investigated toluene concentrations, the removal efficiency increased as the number of lamps on and, therefore, the ozone supplied to the system increased. The combined process allowed the achievement of higher removal efficiencies. For inlet concentrations of 100–150 ppm the removal resulted higher than 95%, even with only 1 lamp on. In the integrated UV/O_3_ + S process, the elimination capacity with 1 lamp on resulted for all the investigated conditions higher than the elimination of UV/O_3_ process applying 4 lamps illumination. The residual ozone resulting from UV/O_3_ treatment may be easily used for the further degradation of the compounds not completely oxidized in the first phase of the process, since the high solubility in water of the oxidation by-products, ozone included. In that view, the additional wet scrubbing step supported a more sustainable toluene degradation, both from an environmental and economic point of view. It may be possible not only to significantly improve the performances of the process, valorizing the high oxidizing capacity of the hydroxyl radicals released in water, but also to reduce the emission of gaseous by-products into the atmosphere.

## Methods

### Chemicals

Toluene (CAS No 108-88-3) was purchased from Sigma Aldrich with a purity of 99.9% and used for the generation of the synthetic gaseous stream. Potassium iodide (CAS No. 7681-11-0, VMR Chemicals), sulfuric acid (CAS No. 7664-93-9, Carlo Erba), starch paste (CAS No. 9005-84-9, Carlo Erba Reagent) and sodium thiosulfate (CAS No. 10122-17-7, Carlo Erba Reagents) were used for the determination of ozone concentration.

**Figure 6:**
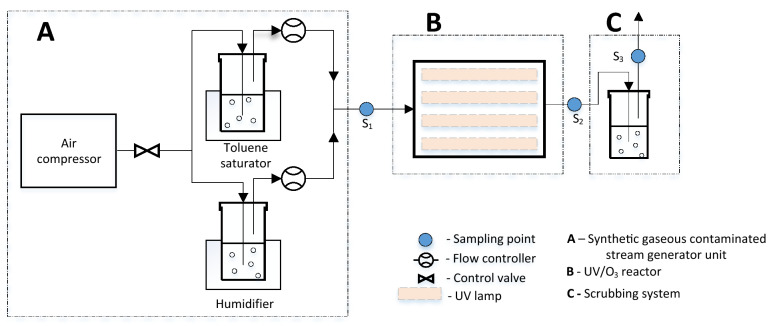
Scheme of the experimental set-up.

Research studies were carried out at the Sanitary Environmental Engineering Division (SEED) Laboratory of the Department of Civil Engineering of the University of Salerno, using a pilot-scale plant composed by a system for the generation of the synthetic gaseous contaminated stream, the UV/O_3_ reactor and the scrubbing unit. Figure 6 shows the scheme of the experimental set-up. Plants or plant parts were not directly involved in the study.

The generation of the synthetic contaminated gaseous stream was obtained by a metered flow of oil-free compressed air forced to pass through a Woulff-bottle pure toluene containing. This concentrated vapor was diluted to the expected concentration with oil-free water-saturated compressed air (Wang et al., 2013). Temperature control was provided by a thermostatic bath maintained at 28 °C ± 10 °C (Lab Instruments). Humidified air and toluene-rich air then merged into a tubing before entering the reactor.

### Experimental set-up

The UV/O_3_ reactor was developed by the SEED research group and consists in a steel photo-reactor composed of a central body (48.6 cm length × 33.7 cm height × 17.8 cm width), which contains four ozone-generating UV lamps (PROCOMAT) characterized by a wavelength of 185 nm, and two pyramidal-truncated hoods (25 cm height). Each lamp has a rating of 14 watts and with a length of 28.7 cm. The working volume of the reactor is to 77˙600 cm^3^. An additional stage of water scrubbing was also investigated. Outlet air from the stainless reactor was forced into 1 L glass reactor with 0.5 L deionized water and distributed by an air diffuser.

### Analytical methods

The analyses were carried out at 3 points, identified as S_1_, S_2_ and S_3_ and representing the inlet and outlet gas streams of the investigated systems.

Two configurations were analyzed for each test, the UV/O_3_ oxidation and the upgraded system with the scrubbing unit. For each configuration, different tests were performed by changing the main operational parameters. The flow rate of humidified air was fixed at 15 Lmin^-1^ and toluene enriched air was varied to achieve the desired inlet concentrations. The minimum inlet concentration was 100 ppm based from the recommended exposure limit established by the National Institute of Occupational Safety and Health^43^. Ozone dosage was varied based on the number of lamps switched on.

Before each test, the reactor and the lamps were cleaned by purging with clean air for 15 min.

Toluene concentration in the gas phase was measured using a GC-PID (Photoionization Detector, Tiger, Ion Science).

Produced ozone and ozone residual from the stand-alone and the combined processes were measured using the Standard Method 2350E 106 (Ozone Demand/Requirement Semi Batch Method).

1 $${\mathrm{O}}_{3}\left(\mathrm{mg}\,{\mathrm{ min}}^{-1}\right)= \frac{\mathrm{V }\cdot \mathrm{ N }\cdot 2400}{\mathrm{t}}$$

where: *V* is the volume of the thiosulfate used for the chemical titration; *N* is the normality; *t* is the time in minutes.

### Performance analysis

Performance studies were carried out analyzing the effects of the variation of the toluene inlet concentration and ozone dosage.

Toluene concentration was varied by adjusting the air flow outlet the toluene evaporator, which enter the reactor. Ozone production resulted dependent on the number of lamps switched on. Each lamp has a potential equal to 34 V. The performances of the investigated processes were evaluated through the following parameters: removal efficiency (RE), elimination capacity (EC) compared to inlet load (IL) and residual ozone (RO).

2 $$RE \left(\%\right)=\frac{{C}_{i}-{C}_{o}}{{C}_{i}}\cdot 100$$

3 $$IL=\frac{Q \cdot {C}_{i}}{V}$$

4 $$EC= \frac{Q ({C}_{i}-{C}_{o})}{V}$$

5 $$RO\left(\%\right)=\frac{{O}_{3,R}}{{O}_{3,P}}\cdot 100$$

where: *Ci* and *Co* denote the inlet and outlet concentrations of toluene in ppm, respectively; *Q* is the air flow rate; *V* is the volume of the reactor; *O*_*3,P*_ and *O*_*3,R*_ are the produced and residual ozone.

## Data Availability

The data elaborated during the current study are available from the corresponding author on reasonable request.
